# Identification of a Potentially Functional circRNA-miRNA-mRNA Regulatory Network in Melanocytes for Investigating Pathogenesis of Vitiligo

**DOI:** 10.3389/fgene.2021.663091

**Published:** 2021-04-21

**Authors:** Lili Li, Zhi Xie, Xiliang Qian, Tai Wang, Minmin Jiang, Jinglin Qin, Chen Wang, Rongqun Wu, Canling Song

**Affiliations:** Department of Dermatology, People’s Hospital of Guangxi Zhuang Autonomous Region, Nanning, China

**Keywords:** vitiligo, circRNAs, ceRNA, metabolism, melanocyte

## Abstract

CircRNAs have been reported to play essential roles in regulating immunity and inflammation, which may be an important regulatory factor in the development of vitiligo. However, the expression profile of circRNAs and their potential biological functions in vitiligo have not been reported so far. In our study we found there are 64 dysregulated circRNAs and 14 dysregulated miRNAs in the patients with vitiligo. Through the correlation analysis, we obtained 12 dysregulated circRNAs and 5 dysregulated miRNAs, forming 48 relationships in the circRNA-miRNA-mRNA regulatory network. Gene Ontology analysis indicated dysregulated circRNAs in vitiligo is closely related to the disorder of the metabolic pathway. The KEGG pathway of dysregulation of circRNAs mainly enriched in the biological processes such as ubiquitin mediated proteolysis, endocytosis and RNA degradation, and in Jak-STAT signaling pathway. Therefore, we found the circRNA-miRNA-mRNA regulatory network are involved in the regulation of numerous melanocyte functions, and these dysregulated circRNAs may closely related to the melanocyte metabolism. Our study provides a theoretical basis for studying the vitiligo pathogenesis from the perspective of circRNA-miRNA-mRNA network.

## Introduction

Vitiligo is characterized by the progressive disappearance of melanocytes, resulting in depigmentation of the skin and/or hair (Malhotra and Dytoc, 2013). The exact pathogenesis remains unknown. Several hypotheses about the pathogenesis mechanisms have been implicated, including genetic, autoimmune, biochemical factors, oxidative stress, and neural or viral causes (Iannella et al., 2016).

Circular RNAs (circRNAs) are a type of covalently closed, single-stranded circular non-coding RNA (Chen and Yang, 2015). Accumulating evidence has shown that circRNAs are widespread and diverse throughout eukaryotic cells and that they have multiple biological functions and play potentially important roles in various diseases (Lei et al., 2018). CircRNAs play an important role in regulating cell proliferation, inflammation and immunity (Chen et al., 2019b; Liu et al., 2019). Therefore, circRNAs are considered to be an important regulator of the onset and development of vitiligo and may be a new therapeutic target for vitiligo.

Circular RNAs are rich in miRNA binding sites (miRNA response elements, MREs), serving as miRNA sponges or competitive endogenous RNAs (ceRNAs). CircRNA regulates mRNA at the post-transcriptional level by completely binding to shared miRNAs (Kristensen et al., 2019). However, circRNAs as ceRNAs mediating pathological processes has not been reported in vitiligo.

Studies on circRNA regulation in vitiligo are limited, the expression profile of circRNAs and their potential biological functions in vitiligo have not been reported so far. In our study, we used RNA-seq to investigated comprehensive circRNA expression profile between vitiligo lesions and normal healthy skin tissue, further to explore the possible regulated mechanism of circRNAs in the pathogenesis of vitiligo.

## Materials and Methods

### Patients and Tissue Specimens

Fresh tissues were obtained from patients with vitiligo and normal donors, from April 2019 to April 2020. The study was approved by the committee of People’s Hospital of Guangxi Zhuang Autonomous Region and all subjects signed an informed consent form under the code KY-ZGR-2019-013. This study included a total of 6 adult patients and 6 control subjects, male and female adults between the ages of 30–60. The patients with vitiligo were diagnosed with vitiligo vulgaris, defined as a condition with depigmentation between 10% and 80% of the body surface. The detail information was shown in Supplementary Table 1.

Skin tissue obtained by skin punch biopsy (4 mm diameter) of the normal areas from normal donors and the lesions from vitiligo patients. Two skin biopsies were obtained from each patient with vitiligo and control subjects. The biopsy was obtained from the central part of the affected skin from patients with vitiligo. Control skin biopsy was taken from healthy control subjects at the same part vs. patient with vitiligo.

### RNA Extraction and Qualification?

Total RNA was extracted from each skin biopsies by RNAprep Pure Tissue Kit (TIANGEN BIOTECH, Beijing, China) in accordance with the manufacturer’s instructions.

We monitored RNA degradation and contamination used 1% agarose gels. And using the NanoPhotometer^®^, spectrophotometer (IMPLEN, CA, United States) to check RNA purity. Then we measured RNA concentration by Qubit^®^, RNA Assay Kit in Qubit^®^, 2.0 Flurometer (Life Technologies, CA, United States) and assessed RNA integrity by the RNA Nano 6000 Assay Kit of the Bioanalyzer 2100 system (Agilent Technologies, CA, United States).

### RNA-Seq

Details of the circRNA-seq and miRNA-seq methods are described in Supplementary Materials. Briefly describe as that:

#### CircRNA-Seq

A total amount of 3 μg RNA per sample was used as input material for the RNA sample preparations. Sequencing libraries were generated using NEBNext^®^, UltraTM RNA Library Prep Kit for Illumina^®^, (NEB, United States) following manufacturer’s recommendations and index codes were added to attribute sequences to each sample. Briefly, mRNA was purified from total RNA using poly-T oligo-attached magnetic beads. In order to select cDNA fragments of preferentially 150∼200 bp in length, the library fragments were purified with AMPure XP system (Beckman Coulter, Beverly, United States). Then 3 μl USER Enzyme (NEB, United States) was used with size-selected, adaptor-ligated cDNA at 37°C for 15 min followed by 5 min at 95°C before PCR. Then PCR was performed with Phusion High-Fidelity DNA polymerase, Universal PCR primers and Index (X) Primer. At last, PCR products were purified (AMPure XP system) and library quality was assessed on the Agilent Bioanalyzer 2100 system. The clustering of the index-coded samples was performed on a cBot Cluster Generation System using TruSeq PE Cluster Kit v3-cBot-HS (Illumia) according to the manufacturer’s instructions. After cluster generation, the library preparations were sequenced on an Illumina Hiseq platform and 125 bp/150 bp paired-end reads were generated. Index of the reference genome was built using STAR and paired-end clean reads were aligned to the reference genome using STAR (v2.5.1b). The circRNA were detected and identified using find_circ and CIRI2 (Memczak et al., 2013; Gao et al., 2018). HTSeq v0.6.0 was used to count the reads numbers mapped to each gene. Differential expression analysis of two conditions/groups (two biological replicates per condition) was performed using the DESeq2 R package (1.10.1). Genes with an adjusted *P*-value < 0.05 found by DESeq2 were assigned as differentially expressed.

#### MiRNA-Seq

A total amount of 3 μg total RNA per sample was used as input material for the small RNA library. Sequencing libraries were generated using NEBNext^®^, Multiplex Small RNA Library Prep Set for Illumina^®^, (NEB, United States) following manufacturer’s recommendations and index codes were added to attribute sequences to each sample. Library quality was assessed on the Agilent Bioanalyzer 2100 system using DNA High Sensitivity Chips. The clustering of the index-coded samples was performed on a cBot Cluster Generation System using TruSeq SR Cluster Kit v3-cBot-HS (Illumia) according to the manufacturer’s instructions. After cluster generation, the library preparations were sequenced on an Illumina Hiseq 2500/2000 platform and 50 bp single-end reads were generated. The small RNA tags were mapped to reference sequence by Bowtie without mismatch to analyze their expression and distribution on the reference (Langmead et al., 2009). miRBase20.0 was used as reference, modified software mirdeep2 and srna-tools-cli were used to obtain the potential miRNA and draw the secondary structures. To remove tags originating from protein-coding genes, repeat sequences, rRNA, tRNA, snRNA, and snoRNA, small RNA tags were mapped to RepeatMasker, Rfam database or those types of data from the specified species itself. The characteristics of hairpin structure of miRNA precursor can be used to predict novel miRNA. The available software miREvo and mirdeep2 were integrated to predict novel miRNA through exploring the secondary structure, the Dicer cleavage site and the minimum free energy of the small RNA tags unannotated in the former steps. miRNA expression levels were estimated by TPM (transcript per million) through the following criteria: Normalization formula: Normalized expression = mapped readcount/Total reads^∗^1000000. Differential expression analysis of two conditions/groups was performed using the DESeq R package (1.8.3). The *P*-values was adjusted using the Benjamini and Hochberg method. Corrected *P*-value of 0.05 was set as the threshold for significantly differential expression by default.

### Real-Time qPCR

To validate the RNA-Seq data, we randomly selected 2 of circRNA and 2 of miRNA for qRT-PCR analysis. Total RNA was extracted from each skin tissue sample, and then reverse-transcribed into cDNA using PrimeScript^TM^ RT reagent Kit with gDNA Eraser (Takara, Dalian, China) according to the manufacturer’s instruction.

Quantitative PCR (qPCR) was performed using the SYBR^®^, Premix Ex TaqTM II (Tli RNase H Plus) Kit with a Bio-Rad CFX Manager 3.1 real-time PCR system (CFX96^TM^ Real-Time PCR, Bio-Rad, United States). The circRNA expression were normalized to GAPDH as an endogenous reference transcript, the miRNA expression were normalized to that of U6 (Zhong et al., 2019). The relative expression levels of circRNA and miRNA were calculated using the 2^–Δ^ ^Δ^ ^*Ct*^ method. The specific primers for each gene are listed in Supplementary Table 2. Data shown represent the means of 3 experiments.

### GO Annotations and KEGG Pathway Analyses

Gene Ontology (GO) enrichment analysis of differentially expressed genes was implemented by the clusterProfiler R package, in which gene length bias was corrected. GO terms with corrected *P*-value less than 0.05 were considered significantly enriched by differential expressed genes.

KEGG is a database resource for understanding high-level functions and utilities of the biological system, such as the cell, the organism and the ecosystem, from molecular-level information, especially large-scale molecular datasets generated by genome sequencing and other high-through put experimental technologies^1^. We used clusterProfiler R package to test the statistical enrichment of differential expression genes in KEGG pathways.

### Annotation for circRNA-miRNA-mRNA Interaction

Predicting circRNA-miRNA interactions and the target gene of miRNA were performed by TargetScan^2^ and miRanda^3^. Upregulation and downregulation of circRNA and miRNA were identified. Then, the circRNA-ceRNA regulatory networks were constructed through the Cytoscape software V2.7.0 (San Diego, CA, United States).

### Statistical Analysis

Statistical analyses were performed using SPSS v16.0 software (SPSS, Inc., Chicago, IL, United States). All data were expressed as the mean ± SEM. *p* < 0.05 was statistically significant.

## Results

### Overview of circRNA-Seq Data

A total of 583,336,760 raw reads were generated, 298,292,356 for patients with vitiligo, and 285,044,404 for control subjects. After poly(N)-containing, low-quality, and adaptor-containing reads were removed from the raw data, 572,982,714 clean reads remained: 293,183,756 for patients and 279,798,958 for control subjects. The clean reads were mapped to the available reference sequence using Hisat2^4^ (Pertea et al., 2016). circRNAs were detected and identified using find_circ and CIRI, 3,633 circRNAs were detected (Memczak et al., 2013; Gao et al., 2015). These circRNAs were used for subsequent analyses.

### Overview of miRNA-Seq Data

A total of 71,225,504 raw reads were generated, 35,549,729 for patients with vitiligo, and 35,675,775 for control subjects. After removal of low-quality and adaptor sequences, 69,812,665 clean reads remained: 34,804,147 for patients and 35,008,518 for control subjects. Further, we integrated miREvo and miRDeep2 software to predict previously unidentified miRNAs. Finally, 1,200 matured miRNAs (1,146 known and 54 novel) were detected. These miRNAs were used for the subsequent analysis.

### Differential Expression of circRNAs and miRNAs in Vitiligo Patients and Control Subjects

**FIGURE 1:**
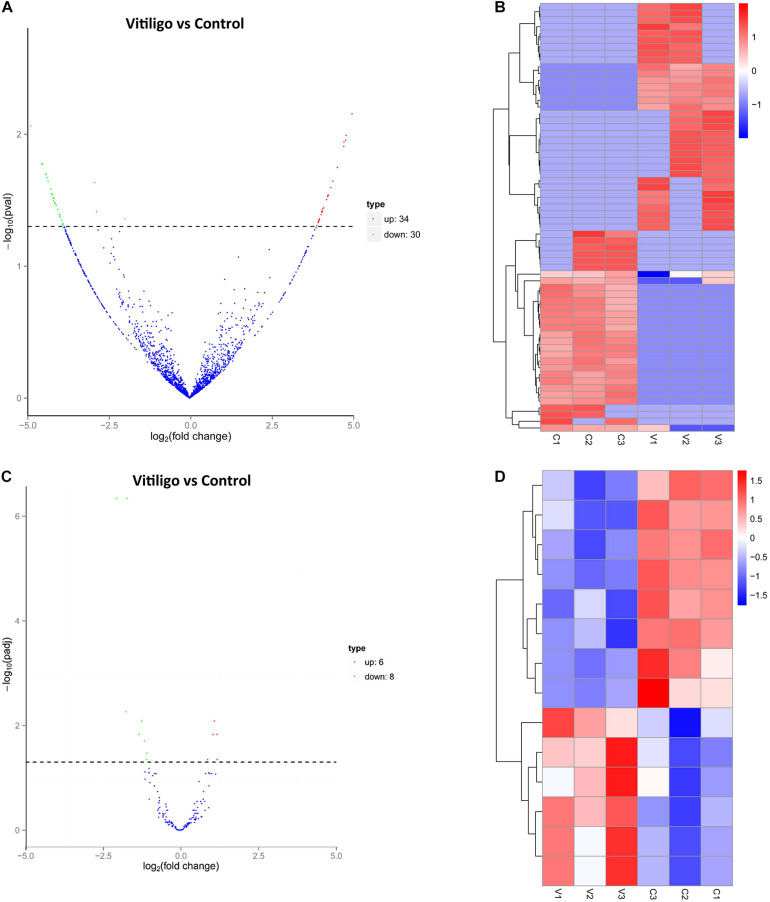
Expression Profiles of dysregulated circRNAs and miRNAs. **(A)** Volcanoplot of circRNAs. Green, red, and blue points represent circRNAs that were downregulated, upregulated, and not significantly different in vitiligo relative to normal control. **(B)** Cluster analysis of expression of circRNAs. Red represent increased expression, blue represent decreased expression. **(C)** Volcanoplot of miRNAs. Green, red and blue points represent miRNAs that were downregulated, upregulated, and not significantly different in vitiligo relative to normal control. **(D)** Cluster analysis of expression of miRNAs. Red represent increased expression, blue represent decreased expression.

We identified 64 significantly dysregulated circRNA transcripts between the two groups in skin tissue of subjects: 34 circRNAs were upregulated and 30 were downregulated in vitiligo patients relative to the control subjects, respectively (Figure 1A and Table 1). We conducted the cluster analysis of the circRNAs expression, and the heatmap results were shown in Figure 1B.

**TABLE 1:** Differently expressed circRNAs in RNA-seq.

**Up-regulated**					
**Name**	***p*-value**	**log2FoldChange**	**circRNA type**	**Chr.**	**Source gene name**

hsa_circ_0003164	0.0020969	5.4848	Exon	14	ZC3H14
hsa_circ_0004276	0.0025305	5.4578	Exon	3	SUCLG2
hsa_circ_0004293	0.003171	5.3221	Exon	11	DDX6
hsa_circ_0005483	0.0069774	4.9833	Exon	16	LMF1
novel_circ_0004933	0.010181	4.8029	Exon	4	GUCY1A1
hsa_circ_0008558	0.0111	4.7886	Exon	16	LONP2
hsa_circ_0007716	0.011469	4.7377	Exon	17	WSB1
hsa_circ_0007803	0.012342	4.7311	Exon	1	LZIC
hsa_circ_0001833	0.017868	4.533	Exon	8	MROH1
novel_circ_0002817	0.022658	4.3978	Exon	1	TNN
hsa_circ_0003770	0.025343	4.3334	Exon	12	AC073610.2;ARF3
hsa_circ_0003060	0.028855	4.253	Exon	3	SUCLG2
hsa_circ_0083265	0.028879	4.253	Exon	8	AC019257.8;ARHGEF10
novel_circ_0001719	0.029043	4.249	Exon	15	IQGAP1
hsa_circ_0001377	0.029173	4.2479	Exon	3	TNK2
hsa_circ_0005133	0.029589	4.2381	Exon	6	DDR1
hsa_circ_0004888	0.031266	4.2048	Exon	2	PPIG
novel_circ_0000033	0.036126	4.1116	Exon	10	SHOC2
novel_circ_0001995	0.036814	4.0813	Intron	17	PAFAH1B1
novel_circ_0004175	0.038492	4.0731	Exon	2	XPO1
novel_circ_0002133	0.038996	4.0627	Exon	17	MBTD1
hsa_circ_0002274	0.038149	4.0575	Exon	1	LPGAT1
novel_circ_0001407	0.040647	4.0352	Exon	14	FOXN3
novel_circ_0004223	0.043796	3.9864	Exon	2	EXOC6B
hsa_circ_0006979	0.044475	3.9757	Exon	16	RSL1D1
novel_circ_0001137	0.044475	3.9757	Exon	13	SETDB2
hsa_circ_0000215	0.044973	3.9668	Intron	10	CAMK1D
hsa_circ_0056838	0.044973	3.9668	Exon	2	7-Mar
hsa_circ_0008378	0.045624	3.9572	Exon	15	PIAS1
hsa_circ_0001699	0.045942	3.9522	Exon	7	CDK13
hsa_circ_0030716	0.046626	3.9423	Exon	13	DOCK9
hsa_circ_0001621	0.049329	3.9029	Exon	6	CASP8AP2
hsa_circ_0006458	0.049329	3.9029	Exon	20	TCFL5
novel_circ_0004181	0.049329	3.9029	Exon	2	EHBP1

**Down-regulated**					

**ID**	***p*-value**	**log2FoldChange**	**circRNA type**	**Chr.**	**Source gene name**

novel_circ_0006548	0.0086321	−4.8793	Exon	9	NIPSNAP3A
hsa_circ_0001789	0.016851	−4.5361	Exon	8	RAB11FIP1
hsa_circ_0012300	0.016611	−4.5127	Exon	1	PIK3R3;AL358075.4
hsa_circ_0087961	0.020012	−4.4137	Intron	9	LPAR1
hsa_circ_0004771	0.019942	−4.4127	Exon	21	NRIP1
hsa_circ_0003572	0.020147	−4.4092	Exon	1	COP1
hsa_circ_0002762	0.021368	−4.3784	Exon	12	CDK17
hsa_circ_0007693	0.022732	−4.3418	Exon	1	ERI3
hsa_circ_0001523	0.025985	−4.2617	Exon	5	ZNF608
hsa_circ_0013252	0.028224	−4.2383	Exon	1	PTBP2
hsa_circ_0023812	0.028701	−4.2025	Exon	11	NARS2
hsa_circ_0077216	0.030247	−4.1728	Exon	6	ME1
hsa_circ_0054877	0.030205	−4.1723	Exon	2	XPO1
hsa_circ_0091669	0.031859	−4.1681	Exon	X	AFF2
hsa_circ_0067985	0.030721	−4.1637	Exon	3	FNDC3B
hsa_circ_0005015	0.032924	−4.1435	Exon	8	HAS2
hsa_circ_0039353	0.033533	−4.1326	Exon	16	CHD9
hsa_circ_0019006	0.034523	−4.0928	Exon	10	WAPL
novel_circ_0005288	0.037215	−4.0444	Exon	5	MRPL22
hsa_circ_0003602	0.040379	−3.9934	Exon	3	SMARCC1
hsa_circ_0085616	0.042635	−3.9764	Exon	8	ASAP1
hsa_circ_0004874	0.041586	−3.9729	Exon	4	SNX25
novel_circ_0005877	0.041578	−3.9717	Exon	6	CASP8AP2
novel_circ_0004624	0.044765	−3.9235	Exon	3	CNOT10
hsa_circ_0020093	0.04839	−3.8923	Exon	10	ATRNL1
hsa_circ_0035649	0.047265	−3.8877	Exon	15	USP3
hsa_circ_0004843	0.048858	−3.8841	Exon	12	BAZ2A
hsa_circ_0028899	0.023216	−2.9137	Exon	12	RNF10
hsa_circ_0003549	0.03858	−2.8558	Exon	4	SEC31A
hsa_circ_0072391	0.043716	−1.9864	Exon	5	HMGCS1

We obtained 14 significantly dysregulated miRNAs: inducing 6 miRNAs were upregulated and 8 were downregulated in skin tissue of vitiligo patients relative to the control subjects (Figure 1C and Table 2). We conducted the cluster analysis of the miRNAs expression, and the heatmap results were shown in Figure 1D.

**TABLE 2:** Differently expressed miRNAs in RNA-seq.

**Up-regulated**		
**Name**	***p*-value**	**log2FoldChange**

hsa-miR-30e-3p	0.0030918	1.198
hsa-miR-27a-3p	0.00058531	1.1978
hsa-miR-24-1-5p	0.00019431	1.109
hsa-miR-218-5p	0.00066766	1.0703
hsa-miR-203b-5p	0.0034802	0.8942
hsa-miR-203a-3p	0.0034927	0.89393

**Down-regulated**		

**Name**	***p*-value**	**log2FoldChange**

hsa-miR-193b-3p	5.19E-09	−2.038
hsa-miR-365a-3p	9.26E-05	−1.7424
hsa-miR-197-3p	5.03E-09	−1.6981
hsa-let-7d-3p	0.00049946	−1.315
hsa-miR-145-5p	0.00023207	−1.2295
hsa-miR-127-3p	0.0010081	−1.1407
hsa-miR-149-5p	0.0035472	−1.0741
hsa-miR-320a-3p	0.0019116	−1.0613

The data showed that these differentially expressed circRNAs were scattered different chromosomes: 34 upregulated circRNAs location in 16 chromosomes, and 30 downregulated circRNAs location in 14 chromosomes. The top chromosomes for the upregulated circRNAs were chr. 2 (6/34), while the top two chromosomes for the downregulated circRNAs were chr. 1 (4/30). The localization of these differentially expressed circRNAs included 32 exonic and 2 intronic in the upregulated circRNAs, and 29 exonic and 1 intronic in the downregulated circRNAs (shown in Table 1).

### Confirmation the Differential Expression of circRNAs and miRNAs

**FIGURE 2:**
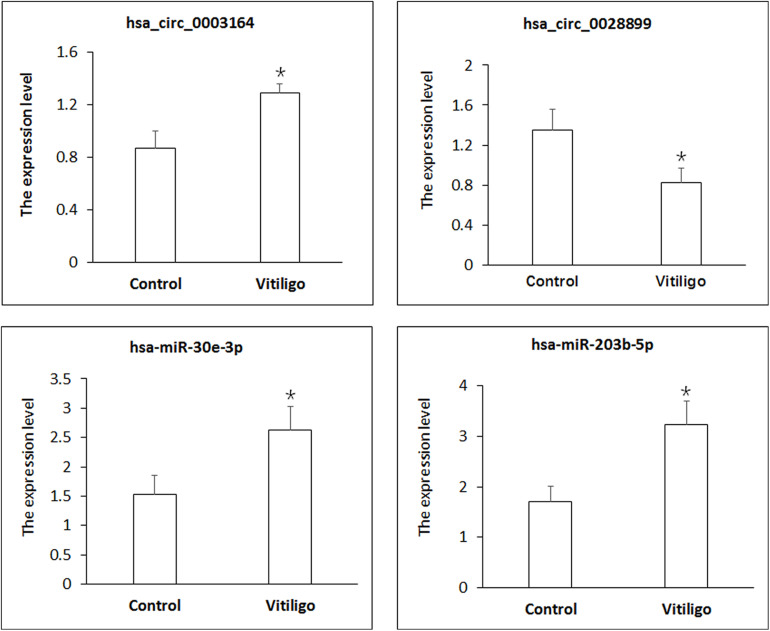
Identification of circRNA and miRNA expression by qPCR. circRNA expression was quantified relative to Gapdh expression level. miRNA expression was quantified relative to U6 expression level. Data are presented as means ± SD (*n* = 3; *p < 0.05).

We sought to confirm the differential expression identified in our RNA-seq experiments using qPCR. The differentially expressed transcripts of circRNAs and miRNAs were randomly selected. As shown in Figure 2, all selected transcripts were detected in skin tissue of vitiligo patients and the control subjects and exhibited significant differential expressions. The expression of hsa_circ_0003164 was 1.29 ± 0.068 in patients with vitiligo vs. 0.86 ± 0.131 in control subjects (*p* < 0.05). The expression of hsa_circ_0028899 was 0.82 ± 0.282 in patients with vitiligo vs. 1.35 ± 0.305 in control subjects (*p* < 0.05). The expression of hsa-miR-30e-3p was 2.62 ± 0.612 in patients with vitiligo vs. 1.52 ± 0.421 in control subjects (*p* < 0.05). The expression of hsa-miR-203b-5p was 3.23 ± 0.673 in patients with vitiligo vs. 1.70 ± 0.409 in control subjects (*p* < 0.05). In summary, our qPCR validation study confirmed the profiles revealed by the RNA-seq data, thus indicating the reliability of the sequencing data.

### Go and KEGG Analyses for the Differential Expressed circRNAs

**FIGURE 3:**
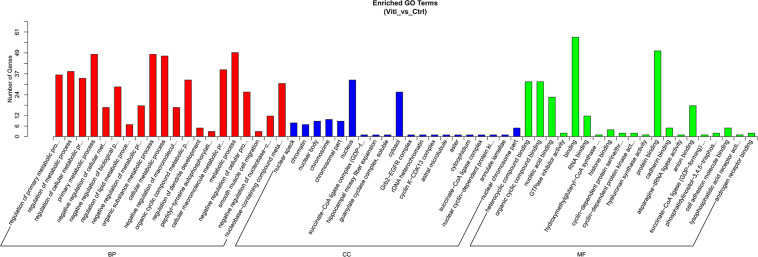
GO Enrichment Annotations of pathological progression of vitiligo. Significantly enriched GO pathways featured *p* values < 0.05.

We performed GO analyses on these dysregulated circRNAs in the networks and the results of the top highly enriched GO terms of cellular component (CC), biological process (BP), and molecular function (MF) are shown in Figure 3. The top terms were molecular function (GO:0003674), cellular process (GO:0009987), and cell (GO:0005623). Several metabolism-associated terms were also observed, such as metabolic process (GO:0008152), organic substance metabolic process (GO:0071704), cellular metabolic process (GO:0044237), regulation of biological process (GO:0050789), cellular macromolecule metabolic process (GO:0044260), and regulation of metabolic process (GO:0019222). Thus, we hypothesis that the pathogenesis of vitiligo may be closely related to many metabolic-related pathways regulated by circRNAs.

**FIGURE 4:**
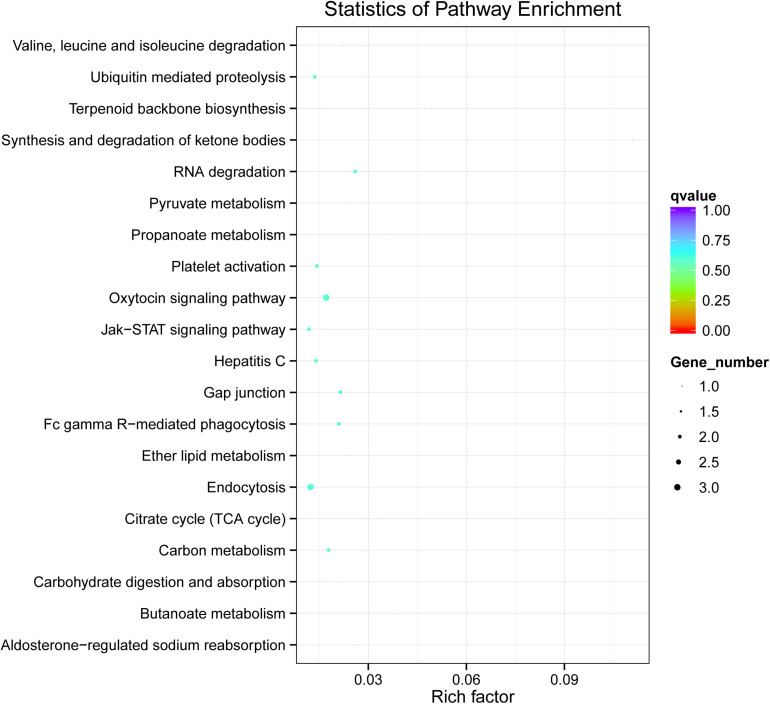
KEGG analysis of circRNA-associated ceRNA network in vitiligo pathology. Significantly enriched KEGG pathways featured *p* values < 0.05.

We further conducted KEGG pathway analysis to determine the signaling cascades related to dysregulated circRNAs by using the *Q*-value scale from 0 to 1, and the top 5 significantly enriched pathways were identified: synthesis and degradation of ketone bodies, terpenoid backbone biosynthesis, butanoate metabolism, propanoate metabolism, and citrate cycle (TCA cycle) (Figure 4).

### Construction of a circRNA-miRNA-mRNA Regulatory Network

**FIGURE 5:**
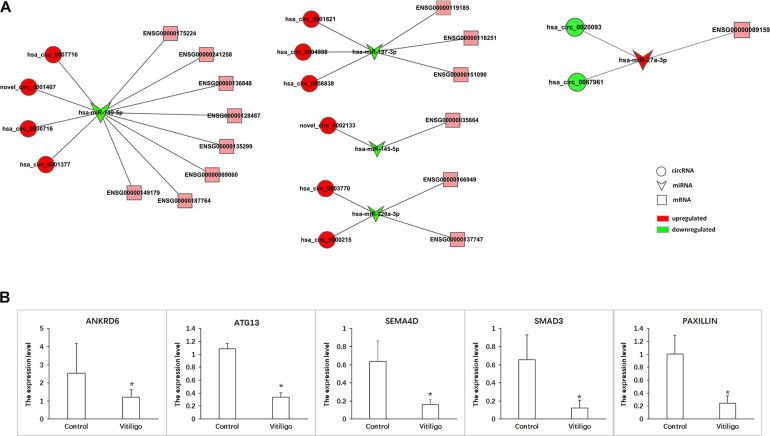
CircRNA-ceRNA networks in vitiligo. **(A)** ceRNA networks were constructed based on identified circRNA-miRNA and miRNA-mRNA interactions. Circle nodes represent circRNAs, triangle nodes represent miRNAs, and rectangle nodes represent mRNAs. Red represent upregulated, green represent downregulated. **(B)** The expression of target gene in networks. Data are presented as means ± SD (*n* = 3; *p < 0.05).

According to ceRNA hypothesis, RNA transcripts effectively communicate with one another. The members of ceRNA compete for the same miRNA response elements (MREs) to regulate one another. We have conducted the regulatory network analysis of circRNA, miRNA, and their target genes. These data show that there are significant differential expression between vitiligo patients and the control subjects (corrected *p* < 0.05). Analysis based on the RNA-seq data, we selected 12 circRNAs and 5 miRNAs that were differentially expressed, containing 48 relationships in the circRNA-miRNA-mRNA regulatory network (Figure 5A and Table 3).

**TABLE 3:** CircRNA-ceRNA networks in vitiligo.

**CircRNA name**	**MiRNA name**	**Gene_id**	**Gene_name**
hsa_circ_0007716	hsa-miR-149-5p	ENSG00000136848	DAB2IP
hsa_circ_0001377	hsa-miR-149-5p	ENSG00000136848	DAB2IP
novel_circ_0001407	hsa-miR-149-5p	ENSG00000136848	DAB2IP
hsa_circ_0030716	hsa-miR-149-5p	ENSG00000136848	DAB2IP
hsa_circ_0007716	hsa-miR-149-5p	ENSG00000187764	SEMA4D
hsa_circ_0001377	hsa-miR-149-5p	ENSG00000187764	SEMA4D
novel_circ_0001407	hsa-miR-149-5p	ENSG00000187764	SEMA4D
hsa_circ_0030716	hsa-miR-149-5p	ENSG00000187764	SEMA4D
hsa_circ_0003770	hsa-miR-320a-3p	ENSG00000137747	TMPRSS13
hsa_circ_0000215	hsa-miR-320a-3p	ENSG00000137747	TMPRSS13
novel_circ_0002133	hsa-miR-145-5p	ENSG00000035664	DAPK2
hsa_circ_0004888	hsa-miR-197-3p	ENSG00000116251	RPL22
hsa_circ_0056838	hsa-miR-197-3p	ENSG00000116251	RPL22
hsa_circ_0001621	hsa-miR-197-3p	ENSG00000116251	RPL22
hsa_circ_0007716	hsa-miR-149-5p	ENSG00000149179	C11orf49
hsa_circ_0001377	hsa-miR-149-5p	ENSG00000149179	C11orf49
novel_circ_0001407	hsa-miR-149-5p	ENSG00000149179	C11orf49
hsa_circ_0030716	hsa-miR-149-5p	ENSG00000149179	C11orf49
hsa_circ_0007716	hsa-miR-149-5p	ENSG00000089060	SLC8B1
hsa_circ_0001377	hsa-miR-149-5p	ENSG00000089060	SLC8B1
novel_circ_0001407	hsa-miR-149-5p	ENSG00000089060	SLC8B1
hsa_circ_0030716	hsa-miR-149-5p	ENSG00000089060	SLC8B1
hsa_circ_0003770	hsa-miR-320a-3p	ENSG00000166949	SMAD3
hsa_circ_0000215	hsa-miR-320a-3p	ENSG00000166949	SMAD3
hsa_circ_0004888	hsa-miR-197-3p	ENSG00000151090	THRB
hsa_circ_0056838	hsa-miR-197-3p	ENSG00000151090	THRB
hsa_circ_0001621	hsa-miR-197-3p	ENSG00000151090	THRB
hsa_circ_0007716	hsa-miR-149-5p	ENSG00000241258	CRCP
hsa_circ_0001377	hsa-miR-149-5p	ENSG00000241258	CRCP
novel_circ_0001407	hsa-miR-149-5p	ENSG00000241258	CRCP
hsa_circ_0030716	hsa-miR-149-5p	ENSG00000241258	CRCP
hsa_circ_0007716	hsa-miR-149-5p	ENSG00000128487	SPECC1
hsa_circ_0001377	hsa-miR-149-5p	ENSG00000128487	SPECC1
novel_circ_0001407	hsa-miR-149-5p	ENSG00000128487	SPECC1
hsa_circ_0030716	hsa-miR-149-5p	ENSG00000128487	SPECC1
hsa_circ_0007716	hsa-miR-149-5p	ENSG00000175224	ATG13
hsa_circ_0001377	hsa-miR-149-5p	ENSG00000175224	ATG13
novel_circ_0001407	hsa-miR-149-5p	ENSG00000175224	ATG13
hsa_circ_0030716	hsa-miR-149-5p	ENSG00000175224	ATG13
hsa_circ_0004888	hsa-miR-197-3p	ENSG00000119185	ITGB1BP1
hsa_circ_0056838	hsa-miR-197-3p	ENSG00000119185	ITGB1BP1
hsa_circ_0001621	hsa-miR-197-3p	ENSG00000119185	ITGB1BP1
hsa_circ_0007716	hsa-miR-149-5p	ENSG00000135299	ANKRD6
hsa_circ_0001377	hsa-miR-149-5p	ENSG00000135299	ANKRD6
novel_circ_0001407	hsa-miR-149-5p	ENSG00000135299	ANKRD6
hsa_circ_0030716	hsa-miR-149-5p	ENSG00000135299	ANKRD6
hsa_circ_0020093	hsa-miR-27a-3p	ENSG00000089159	PXN
hsa_circ_0087961	hsa-miR-27a-3p	ENSG00000089159	PXN

The ceRNA network covered two cases: one was circRNA (upregulated in vitiligo)-miRNA (downregulated in vitiligo)-mRNA (upregulated in vitiligo), and the other one was circRNA (downregulated)-miRNA (upregulated)-mRNA (upregulated). Among them, there are 46 relationships in the manner of up-down-up, occupying the leading position, and only 2 relationships in the manner of down-up-down.

These RNA interactions may supply a novel perspective for the pathogenesis of vitiligo. We observed that one miRNA could be regulated by multiple circRNAs, further regulated a variety of downstream genes that are closely related to melanocyte proliferation or melanin synthesis, such as SEMA4D (ENSG00000187764), RPL22 (ENSG00000116251), ATG13 (ENSG00000175224), and ANKRD6 (ENSG00000135299). For example, hsa-miR-149-5p was co-related with hsa_circ_0007716, novel_circ_0001407, hsa_circ_0030716 and hsa_circ_0001377, further to regulate SEMA4D, ATG13, and ANKRD6.

Moreover, it has been reported that ATG13, SEMA4D, SMAD3, and Paxillin are closely related to the function of melanocytes. Therefore, the circRNA-ceRNA networks that regulate these genes may play important roles in regulating the physiology and pathology of vitiligo. These circRNA-ceRNA networks include: hsa_circ_0007716/novel_circ_0001407/hsa_circ_0030716/hsa _circ_0001377 – hsa-miR–149–5p – ENSG00000175224 (ATG13)/ENSG00000187764 (SEMA4D), hsa_circ_0003770/hsa _circ_0000215 – hsa-miR-320-3p – ENSG00000166949 (SMAD3), hsa_circ_0020093/hsa_circ_0087961 – hsa-miR -27a-3p – ENSG00000089159 (PAXILLIN).

The more significant differential expression of circRNA, the more likely it has an important role in the regulatory network. Therefore, we obtained the circRNA-ceRNA networks that might have the important roles in vitiligo pathology: hsa_circ_0007716 – hsa-miR-149-5p – ATG13/SEMA4D, hsa_circ_0003770 –hsa-miR-320a-3p – SMAD3 and hsa_circ_0087961 – hsa-miR-27a-3p - PAXILLIN.

Furthermore, we detected the expression of target genes such ANKRD6, ATG13, SEMA4D, SMAD3, and PAXILLIN to verify that these circRNA-ceRNA networks are involved in the pathological process of vitiligo. As the results shown that the expression of ANKRD6, ATG13, SEMA4D, SMAD3, and PAXILLIN were all significantly downregulated in vitiligo (Figure 5B). And, only the expression change of PAXILLIN conforms to the profile of circRNA-miRNA-mRNA. Therefore, we hypothesize that circ_0087961-miR-27a-3p-PAXILLIN might play an important regulatory role in the pathology of vitiligo.

## Discussion

Vitiligo is a common chronic acquired disease of pigmentation. Recent clinical and experimental studies suggest that the main mechanism leading to vitiligo involves in autoimmune dysfunction, neurotoxic factor destruction, oxidative stress and genetic factors (Iannella et al., 2016). These factors further lead to loss of melanocyte adhesion and destruction of melanocytes, thereby causing pigmentation loss (Speeckaert and van Geel, 2017; Delmas and Larue, 2019). Vitiligo is a complex genetic disease. Fifty genes at least have already been evaluated in order to identify a link with vitiligo (Sharma et al., 2017; Yuan et al., 2019). Recently studies have suggested that the occurrence and progression of vitiligo are due to multiple factors and gene interactions in which non-coding RNAs contribute to an individual’s susceptibility to vitiligo (Mansuri et al., 2016; Vaish et al., 2019).

CircRNAs are non-coding RNAs characterized by a unique covalent closed-loop structure. It is widely known that circRNAs are rich in miRNA binding sites (miRNA response elements, MREs), serving as miRNA sponges or competitive endogenous RNAs (ceRNAs), which leading the inhibitory effect of miRNAs on their target genes and further increased expression of the target genes (Salzman, 2016; Kristensen et al., 2019). In this mechanism the circRNAs acting as miRNA sponges, play an important role in the regulation of diseases via their interaction with disease-associated miRNAs.

In our study we found there are 64 dysregulated circRNAs in the patients with vitiligo, including 34 circRNAs were upregulated and 30 were downregulated. And also there are 14 dysregulated miRNAs identified in vitiligo, 6 miRNAs were upregulated and 8 were downregulated. Through the correlation analysis, we obtained 12 dysregulated circRNAs and 5 dysregulated miRNAs, forming 48 relationships in the circRNA-miRNA-mRNA regulatory network. Our results showed that 12 dysregulated circRNAs had miRNAs binding sites, and were thus predicted to play a regulatory role via the ceRNA mechanism. And these circRNA-miRNA-mRNA regulatory networks may play an important role in the pathological mechanism of vitiligo. For instance, hsa-miR-149-5p targets genes SEMA4D (ENSG00000187764) and ANKRD6 (ENSG00000135299) are involved in regulating melanocyte survival and growth (Soong et al., 2012; Prabhakar et al., 2019). Sema4D is a paracrine factor that protects normal human melanocytes to survive under ultraviolet (UV) radiation. Sema4D could stimulate cell proliferation and regulates the activity of c-Met receptors, which was determined promotes melanocyte migration (Gotte et al., 2007; Soong et al., 2012). Soong et al. (2012) reported that Sema4D as the ligand for Plexin B1 could suppresses c-Met activation and migration and promotes melanocyte survival and growth. Hsa-miR-320a-3p regulated the target gene SMAD3 (ENSG00000166949). Smad3, a signal mediator of the activin/TGF-beta pathway, which associated with microphthalmia-associated transcription factor (MITF) (Funaba et al., 2003; Wehbe et al., 2012). Previous study indicated MITF is a crucial transcription factor expressed in melanocytes (Mizutani et al., 2010; Vachtenheim and Borovansky, 2010; Hu et al., 2020). Hsa-miR-27a-3p targets gene Paxillin (ENSG00000089159). Paxillin, a 69-kDa vinculin binding protein, is significant higher in neonatal melanocytes than in fetal melanocytes (Scott et al., 1995). It indicates that paxillin is closely related to the adhesion ability of melanocytes (Skoczynska et al., 2016).

GO analysis was performed to further annotate the biological functions of the host genes of the differentially expressed circRNAs. Interestingly, the top 20 GO terms of dysregulated circRNAs in vitiligo compared with normal are most enrich in the regulation of primary metabolic process, regulation of cellular metabolic process, negative regulation of cellular metabolic process, primary metabolic process, organic substance metabolic process, cellular metabolic process, organic cyclic compound metabolic process and cellular macromolecule metabolic process (Pietrzak et al., 2012; Atas and Gonul, 2017; Sahoo et al., 2017). This indicates that the dysregulation of circRNAs is closely related to the disorder of the metabolic pathway. In addition, the GO analysis of miRNA also showed that dysregulated miRNAs are closely related to biological or cellular metabolism (Supplementary Figure 1A).

The KEGG pathway of dysregulation of circRNAs mainly enriched in the biological processes such as ubiquitin mediated proteolysis, endocytosis and RNA degradation, and in Jak-STAT signaling pathway. Some studies have indicated that the Jak-STAT signaling pathway is involved in the immune-mediated inflammatory skin diseases such as vitiligo (Chen et al., 2019a; Gomez-Garcia et al., 2019; Montilla et al., 2019; Samaka et al., 2019). Furthermore, through KEGG analysis of miRNAs, some miRNA dysregulated lead to the disorder of certain signaling pathways (Supplementary Figure 1B), such as p53 signaling pathway, mTOR signaling pathway, Insulin signaling pathway and HIF-1 signaling pathway. Studies have shown that p53 signaling pathway and mTOR signaling pathway all played an important role in the synthesis or metabolism of melanin (Kadekaro et al., 2012; Chang et al., 2017; Yang et al., 2018; Li et al., 2019).

We elucidated the circRNA-associated-ceRNA profiles of vitiligo using deep RNA-seq analysis. From the analysis results, we found the circRNA-miRNA-mRNA regulatory network are involved in the regulation of numerous melanocyte functions, further GO and KEGG analysis show that the regulatory functions of these dysregulated circRNAs may closely related to the melanocyte metabolism. Through the analysis of the circRNA-miRNA-mRNA network, we found circ_0087961-miR-27a-3p-PAXILLIN might play an important regulatory role in the pathology of vitiligo. Our study provides a theoretical basis for studying the vitiligo pathogenesis from the perspective of circRNA-miRNA-mRNA network.

## Data Availability Statement

The data presented in the study are deposited in the NCBI repository (https://www.ncbi.nlm.nih.gov/sra/PRJNA712982), accession number PRJNA712982.

## Ethics Statement

The studies involving human participants were reviewed and approved by People’s Hospital of Guangxi Zhuang Autonomous Region. The patients/participants provided their written informed consent to participate in this study.

## Author Contributions

LL conceived and wrote the article. LL and ZX designed and performed the research. XQ, TW, MJ, and JQ analyzed the data. CW, RW, and CS contributed essential reagents or tools. All authors read and approved the manuscript.

## Conflict of Interest

The authors declare that the research was conducted in the absence of any commercial or financial relationships that could be construed as a potential conflict of interest.

## Footnotes

1 http://www.genome.jp/kegg/

2 http://www.targetscan.org

3 http://www.microrna

4 http://ccb.jhu.edu/software/hisat2

## References

[B1] Atas H. Gonul M. (2017). Increased risk of metabolic syndrome in patients with vitiligo. *Balkan Med. J.* 34 219–225. 10.4274/balkanmedj.2016.1005 28443562 PMC5450861

[B2] Chang C. H. Kuo C. J. Ito T. Su Y. Y. Jiang S. T. Chiu M. H. (2017). CK1alpha ablation in keratinocytes induces p53-dependent, sunburn-protective skin hyperpigmentation. *Proc. Natl. Acad. Sci. U.S.A.* 114 E8035–E8044. 28878021 10.1073/pnas.1702763114 PMC5617257

[B3] Chen L. L. Yang L. (2015). Regulation of circRNA biogenesis. *RNA Biol.* 12 381–388. 10.1080/15476286.2015.1020271 25746834 PMC4615371

[B4] Chen X. Guo W. Chang Y. Chen J. Kang P. Yi X. (2019a). Oxidative stress-induced IL-15 trans-presentation in keratinocytes contributes to CD8(+) T cells activation via JAK-STAT pathway in vitiligo. *Free Radic. Biol. Med.* 139 80–91. 10.1016/j.freeradbiomed.2019.05.011 31078730

[B5] Chen X. Yang T. Wang W. Xi W. Zhang T. Li Q. (2019b). Circular RNAs in immune responses and immune diseases. *Theranostics* 9 588–607. 10.7150/thno.29678 30809295 PMC6376182

[B6] Delmas V. Larue L. (2019). Molecular and cellular basis of depigmentation in vitiligo patients. *Exp. Dermatol.* 28 662–666. 10.1111/exd.13858 30536790

[B7] Funaba M. Ikeda T. Murakami M. Ogawa K. Tsuchida K. Sugino H. (2003). Transcriptional activation of mouse mast cell Protease-7 by activin and transforming growth factor-beta is inhibited by microphthalmia-associated transcription factor. *J. Biol. Chem.* 278 52032–52041. 10.1074/jbc.m306991200 14527958

[B8] Gao Y. Wang J. Zhao F. (2015). CIRI: an efficient and unbiased algorithm for de novo circular RNA identification. *Genome Biol.* 16:4. 10.1186/s13059-014-0571-3 PMC4316645 25583365

[B9] Gao Y. Zhang J. Zhao F. (2018). Circular RNA identification based on multiple seed matching. *Brief. Bioinform.* 19 803–810. 10.1093/bib/bbx014 28334140

[B10] Gomez-Garcia F. Gomez-Arias P. J. Hernandez J. Montilla A. M. Gay-Mimbrera J. Aguilar-Luque M. (2019). Drugs targeting the JAK/STAT pathway for the treatment of immune-mediated inflammatory skin diseases: protocol for a scoping review. *BMJ Open* 9:e028303. 10.1136/bmjopen-2018-028303 PMC6538201 31122999

[B11] Gotte M. Kersting C. Radke I. Kiesel L. Wulfing P. (2007). An expression signature of syndecan-1 (CD138), E-cadherin and c-met is associated with factors of angiogenesis and lymphangiogenesis in ductal breast carcinoma in situ. *Breast Cancer Res.* 9:R8. 10.1186/bcr1641 PMC1851383 17244359

[B12] Hu M. Chen C. Liu J. Cai L. Shao J. Chen Z. (2020). The melanogenic effects and underlying mechanism of paeoniflorin in human melanocytes and vitiligo mice. *Fitoterapia* 140:104416. 10.1016/j.fitote.2019.104416 31704261

[B13] Iannella G. Greco A. Didona D. Didona B. Granata G. Manno A. (2016). Vitiligo: pathogenesis, clinical variants and treatment approaches. *Autoimmun. Rev.* 15 335–343. 10.1016/j.autrev.2015.12.006 26724277

[B14] Kadekaro A. L. Chen J. Yang J. Chen S. Jameson J. Swope V. B. (2012). Alpha-melanocyte-stimulating hormone suppresses oxidative stress through a p53-mediated signaling pathway in human melanocytes. *Mol. Cancer Res.* 10 778–786. 10.1158/1541-7786.mcr-11-0436 22622028

[B15] Kristensen L. S. Andersen M. S. Stagsted L. V. W. Ebbesen K. K. Hansen T. B. Kjems J. (2019). The biogenesis, biology and characterization of circular RNAs. *Nat. Rev. Genet.* 20 675–691. 31395983 10.1038/s41576-019-0158-7

[B16] Langmead B. Trapnell C. Pop M. Salzberg S. L. (2009). Ultrafast and memory-efficient alignment of short DNA sequences to the human genome. *Genome Biol.* 10:R25. 10.1186/gb-2009-10-3-r25 PMC2690996 19261174

[B17] Lei K. Bai H. Wei Z. Xie C. Wang J. Li J. (2018). The mechanism and function of circular RNAs in human diseases. *Exp. Cell Res.* 368 147–158. 10.1016/j.yexcr.2018.05.002 29730164

[B18] Li C. Chen H. Lan Z. He S. Chen R. Wang F. (2019). mTOR-dependent upregulation of xCT blocks melanin synthesis and promotes tumorigenesis. *Cell Death Differ.* 26 2015–2028. 10.1038/s41418-019-0274-0 30760873 PMC6748149

[B19] Liu C. X. Li X. Nan F. Jiang S. Gao X. Guo S. K. (2019). Structure and degradation of circular RNAs regulate PKR activation in innate immunity. *Cell* 177 865.e21–880.e21. 31031002 10.1016/j.cell.2019.03.046

[B20] Malhotra N. Dytoc M. (2013). The pathogenesis of vitiligo. *J. Cutan. Med. Surg*. 17 153–172. 23673299 10.2310/7750.2012.12005

[B21] Mansuri M. S. Singh M. Begum R. (2016). miRNA signatures and transcriptional regulation of their target genes in vitiligo. *J. Dermatol. Sci.* 84 50–58. 10.1016/j.jdermsci.2016.07.003 27450903

[B22] Memczak S. Jens M. Elefsinioti A. Torti F. Krueger J. Rybak A. (2013). Circular RNAs are a large class of animal RNAs with regulatory potency. *Nature* 495 333–338. 10.1038/nature11928 23446348

[B23] Mizutani Y. Hayashi N. Kawashima M. Imokawa G. (2010). A single UVB exposure increases the expression of functional KIT in human melanocytes by up-regulating MITF expression through the phosphorylation of p38/CREB. *Arch. Dermatol. Res.* 302 283–294. 10.1007/s00403-009-1007-x 19937254

[B24] Montilla A. M. Gomez-Garcia F. Gomez-Arias P. J. Gay-Mimbrera J. Hernandez-Parada J. Isla-Tejera B. (2019). Scoping review on the use of drugs targeting JAK/STAT pathway in atopic dermatitis, vitiligo, and alopecia areata. *Dermatol. Therapy* 9 655–683. 10.1007/s13555-019-00329-y PMC6828894 31606872

[B25] Pertea M. Kim D. Pertea G. M. Leek J. T. Salzberg S. L. (2016). Transcript-level expression analysis of RNA-seq experiments with HISAT, StringTie and Ballgown. *Nat. Protoc.* 11 1650–1667. 10.1038/nprot.2016.095 27560171 PMC5032908

[B26] Pietrzak A. Bartosinska J. Hercogova J. Lotti T. M. Chodorowska G. (2012). Metabolic syndrome in vitiligo. *Dermatol. Therapy* 25 (Suppl. 1), S41–S43. 10.1111/dth.12012 23237037

[B27] Prabhakar K. Rodriotaguez C. I. Jayanthy A. S. Mikheil D. M. Bhasker A. I. Perera R. J. (2019). Role of miR-214 in regulation of beta-catenin and the malignant phenotype of melanoma. *Mol. Carcinogenesis.* 58 1974–1984. 10.1002/mc.23089 PMC6800786 31338875

[B28] Sahoo A. Lee B. Boniface K. Seneschal J. Sahoo S. K. Seki T. (2017). MicroRNA-211 regulates oxidative phosphorylation and energy metabolism in human vitiligo. *J. Invest. Dermatol.* 137 1965–1974. 10.1016/j.jid.2017.04.025 28502800 PMC6233982

[B29] Salzman J. (2016). Circular RNA expression: its potential regulation and function. *Trends Genet.* 32 309–316. 10.1016/j.tig.2016.03.002 27050930 PMC4948998

[B30] Samaka R. M. Basha M. A. Menesy D. (2019). Role of Janus kinase 1 and signal transducer and activator of transcription 3 in vitiligo. *Clin. Cosmet. Investig. Dermatol.* 12 469–480. 10.2147/ccid.s210106 PMC6612046 31303777

[B31] Scott G. A. Liang H. Cassidy L. L. (1995). Developmental regulation of focal contact protein expression in human melanocytes. *Pigment Cell Res.* 8 221–228. 10.1111/j.1600-0749.1995.tb00667.x 8610074

[B32] Sharma C. K. Sharma M. Prasad K. (2017). Involvement of different genes expressions during immunological and inflammatory responses in vitiligo. *Crit. Rev. Eukaryot. Gene Exp.* 27 277–287. 10.1615/critreveukaryotgeneexpr.2017019558 29199612

[B33] Skoczynska A. Budzisz E. Podgorna K. Rotsztejn H. (2016). Paxillin and its role in the aging process of skin cells. *Postepy Hig. Med. Dosw.* 70 1087–1094. 10.5604/17322693.1221385 27708212

[B34] Soong J. Chen Y. Shustef E. M. Scott G. A. (2012). Sema4D, the ligand for Plexin B1, suppresses c-Met activation and migration and promotes melanocyte survival and growth. *J. Investig. Dermatol.* 132 1230–1238. 10.1038/jid.2011.414 22189792 PMC3305852

[B35] Speeckaert R. van Geel N. (2017). Vitiligo: an update on pathophysiology and treatment options. *Am. J. Clin. Dermatol.* 18 733–744. 10.1007/s40257-017-0298-5 28577207

[B36] Vachtenheim J. Borovansky J. (2010). “Transcription physiology” of pigment formation in melanocytes: central role of MITF. *Exp. Dermatol.* 19 617–627. 10.1111/j.1600-0625.2009.01053.x 20201954

[B37] Vaish U. Kumar A. A. Varshney S. Ghosh S. Sengupta S. Sood C. (2019). Micro RNAs upregulated in Vitiligo skin play an important role in its aetiopathogenesis by altering TRP1 expression and keratinocyte-melanocytes cross-talk. *Sci. Rep.* 9:10079. 10.1038/s41598-019-46529-6 PMC6625998 31300697

[B38] Wehbe M. Soudja S. M. Mas A. Chasson L. Guinamard R. de Tenbossche C. P. (2012). Epithelial-mesenchymal-transition-like and TGFbeta pathways associated with autochthonous inflammatory melanoma development in mice. *PLoS One* 7:e49419. 10.1371/journal.pone.0049419 PMC3500287 23173060

[B39] Yang Z. Zeng B. Pan Y. Huang P. Wang C. (2018). Autophagy participates in isoliquiritigenin-induced melanin degradation in human epidermal keratinocytes through PI3K/AKT/mTOR signaling. *Biomed. Pharmacother.* 97 248–254. 10.1016/j.biopha.2017.10.070 29091873

[B40] Yuan X. Meng D. Cao P. Sun L. Pang Y. Li Y. (2019). Identification of pathogenic genes and transcription factors in vitiligo. *Dermatol. Therapy* 32:e13025. 10.1111/dth.13025 31306558

[B41] Zhong S. Zhou S. Yang S. Yu X. Xu H. Wang J. (2019). Identification of internal control genes for circular RNAs. *Biotechnol. Lett.* 41 1111–1119. 10.1007/s10529-019-02723-0 31428905

