# Supplementary figures and images for: Identification of a Potentially Functional circRNA-miRNA-mRNA Regulatory Network in Melanocytes for Investigating Pathogenesis of Vitiligo
Source: Front Genet. 2021 Apr 21;12:663091. doi: 10.3389/fgene.2021.663091 (PMC8098995; doi:10.3389/fgene.2021.663091)

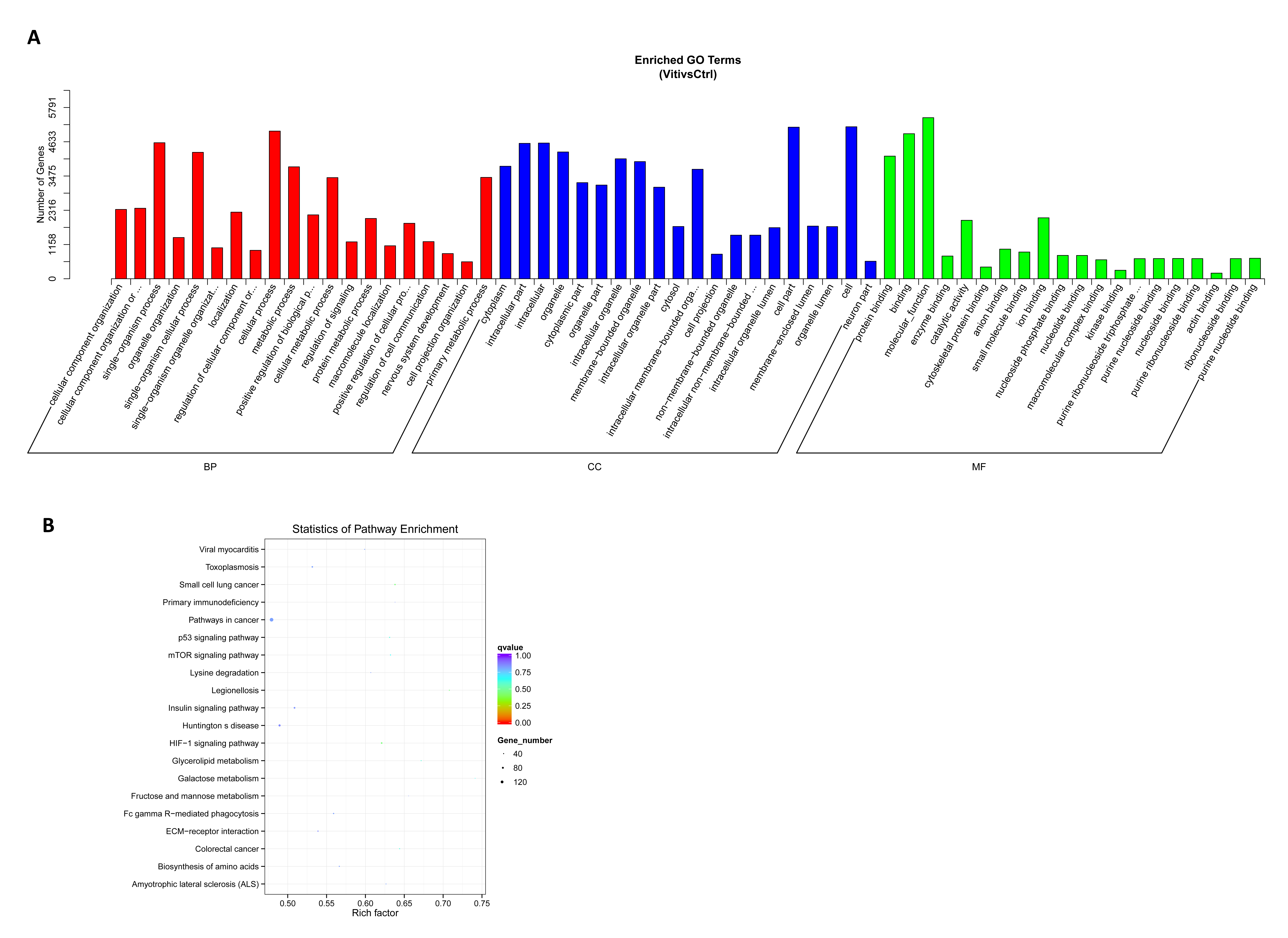

Supplement: Supplementary file 1 [file Image_1.tif]
