# Supplementary material for: Identification of a Potentially Functional circRNA-miRNA-mRNA Regulatory Network in Melanocytes for Investigating Pathogenesis of Vitiligo
Source: Front Genet. 2021 Apr 21;12:663091. doi: 10.3389/fgene.2021.663091 (PMC8098995; doi:10.3389/fgene.2021.663091)
Supplement: Supplementary file 3 [file Table_1.docx]

**Table S1. Clinical characteristics of patients with vitiligo.**

| Case | Aged | Sex | Subtypes of vitiligo | Disease activity | Pathological grade | Involved site |
| --- | --- | --- | --- | --- | --- | --- |
| 1 | 46 | female | Nonsegmental | progressive stage | 2 | left upper limb |
| 2 | 32 | female | Nonsegmental | stable phase | 3 | trunk |
| 3 | 38 | female | Nonsegmental | progressive stage | 3 | left lateral limb |
| 4 | 38 | male | Nonsegmental | progressive stage | 3 | abdomen |
| 5 | 45 | male | Nonsegmental | stable phase | 4 | left upper limb |
| 6 | 34 | female | Nonsegmental | progressive stage | 3 | right upper limb |
