# Supplementary material for: Identification of a Potentially Functional circRNA-miRNA-mRNA Regulatory Network in Melanocytes for Investigating Pathogenesis of Vitiligo
Source: Front Genet. 2021 Apr 21;12:663091. doi: 10.3389/fgene.2021.663091 (PMC8098995; doi:10.3389/fgene.2021.663091)
Supplement: Supplementary file 4 [file Table_2.docx]

**Table S2. Primers used in qPCR analysis**

| **Acession No.** | **Primer sequence (5‘-3’)** | **Primer direction** |
| --- | --- | --- |
| hsa_circ_0003164 | AGCAGTGTGTCTGTGCCTGC | forward primer |
|  | CGAGTTCTGGGAGCAACTGGA | reverse primer |
| hsa_circ_0028899 | TCTGCTGGGCATGCATCCTG | forward primer |
|  | GCAAAGCCACCAACACCCCT | reverse primer |
| hsa-miR-30e-3p | CTTTCAGTCGGATGTTTACAGC | forward primer |
|  | TGGTGTCGTGGAGTCG | reverse primer |
| hsa-miR-203b-5p | TAGTGGTCCTAAACATTTCACA | forward primer |
|  | TGGTGTCGTGGAGTCG | reverse primer |
| U6 | CAAATTCGTGAAGCGTT | forward primer |
|  | TCACTGGTGTCGTGG | reverse primer |
| GAPDH | GACCTGACCTGCCGTCTA | forward primer |
|  | AGGAGTGGGTGTCGCTGT | reverse primer |
| ATG13 | CTGGGCTTAAGGCGGGAGTG | forward primer |
|  | GCCCCAGACGTTTTGCTCCT | reverse primer |
| SEMA4D | GGGTCCTGGGGCTCATCTCT | forward primer |
|  | TCGCTCTCACCACCGCAATG | reverse primer |
| SMAD3 | CAGCTACCCCGTGCGGAAAC | forward primer |
|  | CAGCAGTGCAGGGTCGGGAA | reverse primer |
| PAXILLIN | AGTTGCGGGGCATAGACGAG | forward primer |
|  | GACTCCAAGTCCGCCAGCAG | reverse primer |
| ANKRD6 | CACAGGTAACCCGCAGGAGC | forward primer |
|  | GACCTTTCTCTCCCGCGCC | reverse primer |
| Actin | TGGCACCCAGCACAATGAA | forward primer |
|  | CTAAGTCAGAGTCCGCCTAGAAGCA | reverse primer |
